# Voluntary Control of Cognitive Activity in Preschool Children: Age-dependent Changes from Ages 3–4 to 4–5

**DOI:** 10.11621/pir.2023.0309

**Published:** 2023-09-30

**Authors:** Marina N. Zakharova, Regina I. Machinskaya

**Affiliations:** a Institute of Developmental Physiology, Moscow, Russia; b Multidiscipline Psychological Center “Territoriya Schast’ya”, Moscow, Russia

**Keywords:** executive functions (EF), working memory, voluntary control, cognitive flexibility, preschool age, neuropsychology, activity theory

## Abstract

**Background:**

Voluntary control of goal-directed behavior and mental activity in preschool children plays a key role in knowledge acquisition and future academic achievement. Studies of voluntary control have mainly concerned 6–8-year old children; much less is known about the ability to exercise voluntary control at early ages. Due to the high prognostic value of the level of development of voluntary control and heterogeneous development of their individual components, it seems actually useful to study age-related changes of these components in children from 3-4 to 4-5 years old.

**Objective:**

To compare age-related changes in executive functions (EF) in children age 3-4 years (mean age: 3.5±0.2 yrs; n = 49; 31 boys) and 4-5 years (mean age: 4.5±0.3 yrs; n = 70; 35 boys).

**Design:**

To assess the different components of EF we used: 1) a qualitative group and individual testing procedure based on the principles of Luria’s theory of the dynamic localization and organization of higher mental functions; and 2) a computerized testing procedure which included the Bourdon-Wiersma cancellation test, the “Hearts and Flowers” conflict test (the Dots task), and the Corsi block-tapping test.

**Results:**

The results showed that different components of voluntary control developed at different rates (heterochronically): there were significant progressive changes from 3-4 to 4-5 years for working memory, assimilation of instructions, switching between separate actions, selective concentration on a target or task, and the distribution of attention. Some other components of EF did not show significant positive dynamics during this period.

**Conclusion:**

The results indicate the importance of applying the activity theory approach to the development of cognitive processes in preschool age.

## Introduction

According to the ideas about the active nature of knowledge acquisition developed by N.F. Talyzina, the formation of concepts is a goal-directed activity, which includes the stages of orientation, execution, control, and correction. This means that not only are specific cognitive abilities (those formed on the basis of a certain subject material) important for learning, but “general activity skills” as well (Talyzina, 1988, p. 40). General activity skills include the ability to plan one’s activities, “meaningful” (voluntary) memorization, and attention. However, voluntary attention isn’t only responsible for developing the capacity to focus; it also enables control.

In neuropsychology and cognitive psychology, different aspects of voluntary control are combined under the umbrella term “executive functions” (EF) (Miyake et al., 2000; Diamond, 2013; Goldstein et al., 2014). Luria’s neuropsychology (Luria, 1973), which is also based on activity approach, allows us to identify and study three components of executive functions: programming, selective regulation, and control.

Various neuropsychological and neurocognitive studies (Garon et al., 2008; Bull et al., 2008; Mozgovye mekhanizmy…, 2014; Alloway, T.P. & Alloway R.G., 2010; Kim et al., 2021) have shown that an important factor in academic performance is not only the effectiveness of EF in the acquisition of school knowledge, but also their maturation at preschool age. In our previously published studies of 5-to-6, and 6-to-7-year-old children, we revealed that children deemed by their teachers to be ready for school learning, were characterized by far more developed EF — including programming, selective regulation, control of mental activity, working memory, inhibitory control, cognitive flexibility, and long-term maintenance of attention — than their peers, who, according to the teachers’ evaluation, could experience difficulties in adapting to school learning (Zakharova et al., 2022).

At the same time, various studies have demonstrated that the state of a child’s EF at an early age is not only an important condition for the success of learning in the future, but is also already an essential factor in the progressive formation of thinking at preschool age. According to studies published by Kharitonova and Munakata (2011), cognitive flexibility (one of the components of EF) in 3-year-old children, is bound up with abstraction skills. Some longitudinal works have shown a linear improvement of voluntary control in 3-to-6 year-old children (Clark et al., 2013; Willoughby et al., 2012).

Taking into consideration the intensive heterochronic morpho-functional maturation of the prefrontal cortex in the given age period (Semenova et al., 2003) on the one hand, and the role of EF in the development of thinking and knowledge acquisition on the other, it was advisable to perform more detailed analysis of age-related changes of separate EF components in preschoolers moving from 3-4 to 4-5 years. The goal of this study was the assessment of the age-related changes in understanding and following instructions, sustained maintenance of the acquired program, and the control of execution of one’s actions, as well as working memory and long-term maintenance of attention during monotonous activity.

## Methods

### Participants

Children age 3–4 (average age — 3.5±0.2 yrs., n = 49, boys = 31, girls = 18) and 4–5 (average age — 4,5±0.3 yrs., n = 70, boys = 35, girls = 35) participated in the current study. All the children attended junior and middle kindergarten groups in Moscow. The children had attended junior groups for at least three months, and middle groups for at least one year. Neither group included children diagnosed with any neurological or mental disorders. Moreover, we excluded five children due to their inability to follow and/or understand task instructions. Informed consent was obtained from the children’s parents after the procedure was explained.

### Procedure

Every child underwent individual assessment, which was divided into 2–3 parts, each performed by the same neuropsychologist. The children age 3–4 were exposed to three sessions of 15–20 minutes each. The children age 4–5 were exposed to two sessions of 25–30 minutes each. If they got tired, the children could take a rest.

### Instruments

The neuropsychological evaluation of the development of the children’s executive functions included two types of analysis. The first was a qualitative assessment of the difficulties and errors in a child’s performance on various neuropsychological tests (The Bourdon–Wiersma Cancellation Test, the Spot the Difference Task, the Reciprocal Motor Programmer Test, the Graphomotor Sequences Task — Repeated Pattern Test, the Maze-tracing Task, and the Digit Symbol Coding Task).

The second involved quantitative measurement of the children’s task performance (rate, accuracy number of errors) on several computerized neurocognitive tests (the Bourdon–Wiersma cancellation test, the “Hearts and Flowers” conflict test, the Dots task, and the Corsi block-tapping test (Akhutina et al., 2019)). The test battery was designed to evaluate the children’s various executive functions: their abilities to follow simple and reversed instructions, to shift from one instruction to another, to maintain programs of task performance (incl. monotonous ones), to inhibit irrelevant responses, and to exercise selective attention and working memory.

### The Data Processing

The qualitative analysis of the neuropsychological probes was based on Luria’s theory of the dynamic localization and organization of higher mental functions (1969). The results of the neuropsychological tests were analyzed by singling out four integral indexes (see *Table 1*), which reflect deficits in the following components of EF (Semenova, 2015): 1) understanding instructions or algorithms of task performance; 2) selective regulation (calculated as the mean score of four different neuropsychological parameters — deficit of overcoming immediate reactions; timely termination of the onset of an action and switching from one action to another one; difficulties in switching from one mode of action to another one, from program to program; and difficulties in sustained maintenance of the acquired program); 3) control of execution of one’s own actions; and 4) the general level of EF development (calculated as the mean score of the three previous neuropsychological indexes).

**Table 1:** Neuropsychological indexes of the deficiency of executive functions

EF components	Deficiency manifestations
1. Understanding instructions or algorithms	Individual features of understanding instructions or algorithms (from the first presentation, after repeated presentation, after joint execution, lack of understanding) of the different tasks: the Cancellation test, Spot the Difference task, Reciprocal Motor Programmer Test, Graphomotor Sequences Test, Maze-tracing Task, and the Digit Symbol Coding Task
2. Selective regulation	
2.1. Overcoming immediate reactions	2.1. Presence of impulsive reactions during the Spot the Difference Task, Cancellation Test, Reciprocal Motor Programmer Test), Graphomotor Sequences Test, the Maze-tracing Task, and Digit Symbol Coding Task
2.2. Switching from one action to another	2.2. Interruptions during the Graphomotor Sequences, echoic reactions under choice Go/no-go task, perseveration under the Digit Symbol Coding Task
2.3. Switching from one mode of action to another, from program to program	2.3. Difficulties in switching between probes in various tasks, and between stimuli during the Reciprocal Motor Programmer Test and Digit Symbol Coding Task
2.4. Sustained maintenance of the acquired program	2.4. Mistakes in following the program (incl. stimuli omission) during the Cancellation Test, Graphomotor Sequences, the Maze-tracing Task, and Digit Symbol Coding Task; program loss during the Spot the Difference task and the Reciprocal Motor Programmer Test
3. Control of execution of one’s own actions	A total of the corrected and uncorrected errors found in all the tasks

When we compared groups, we used the nonparametric Mann–Whitney U test for unrelated samples. The group differences were considered statistically significant, taking into account the Bonferroni correction, at *p* <0.05/8 = 0.006 (the maximum number of pairwise comparisons was 8).

## Results

**Figure 1.**
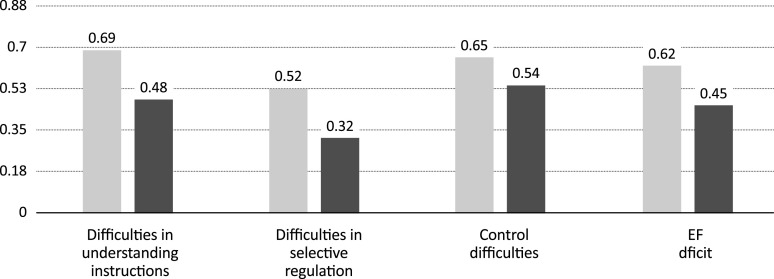
Integral neuropsychological indexes of deficiencies in EF components (higher numbers stand for lower development level)

The comparison of the two preschool groups (see *Figure 1*) revealed lower levels of EF development in children age 3–4 compared to children age 4–5. Consequently, the integral neuropsychological index reflecting EF deficiency (U = 591.0, p < 0.001), and the index reflecting understanding of instructions and algorithms (U = 437.5, p < 0.001), resulted in higher values in the younger group. Selective regulation and control of execution of one’s own actions did not prove to be significantly different in the two given age groups.

Statistically significant differences were also found for one of the parameters included in the index of selective regulation — switching from one program element to another (U = 726.5, p = 0.004).

Computer tests showed a higher deficit in the ability to maintain a program under monotonous tasking in the younger group. The children had been given a computer version of the Cancellation Test presented in two tables. The first table required the child to cross out one target stimulus, and the second table required the child to cross out two target stimuli. It was the last table that revealed the statistically greater number of errors in the younger group (U = 1242.5, p = 0.02).

**Figure 2.**
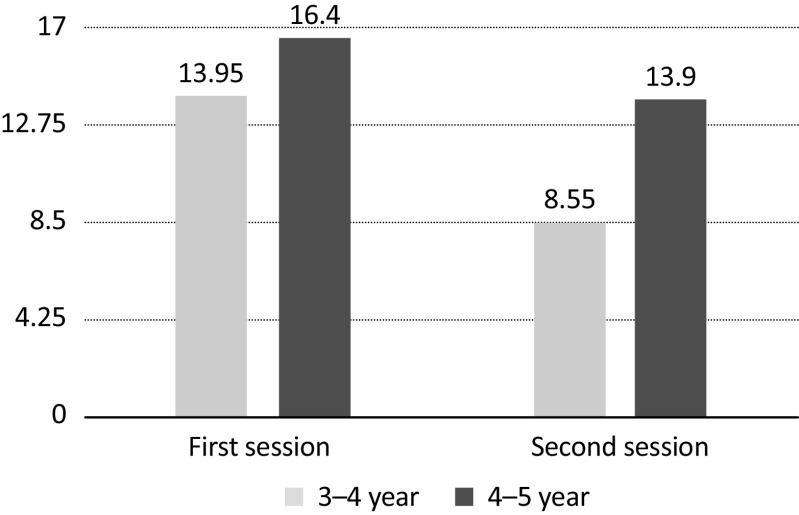
The productivity of the “Hearts and Flowers” conflict test in preschoolers

In the younger group, the computer tests also resulted in a higher score of neuropsychological indexes of deficiency in program switching compared with the older group. While the “Hearts and Flowers” conflict test was underway, intergroup differences proved to be significant for: 1) the productivity of the first (congruent) session performance (U = 728.5, p < 0.0001), as well as the second (incongruent) session performance (U = 483.0, p < 0.0001) (see *Figure 2*); and 2) errors made in the first (U = 741.0, p < 0.0001) and the second (U = 464.5, p < 0.0001) sessions. The third session, which requires switching between two programs, proved to be unsolvable for both groups.

**Figure 3.**
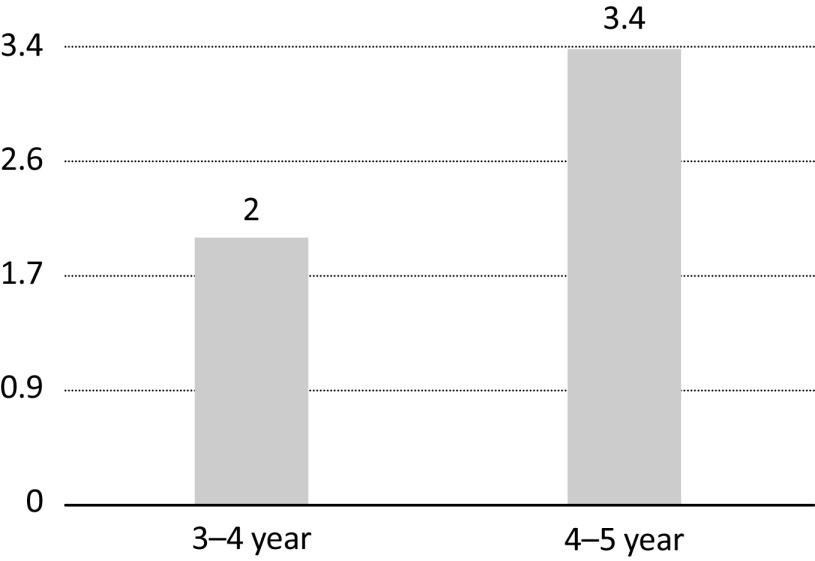
Working memory span in preschoolers

One of the most meaningful experimental probes of our study was a computer version of the Corsi block-tapping test, since it allowed us to assess visual-spatial working memory span (see *Figure 3*). Children age 3–4 were generally good at memorizing a sequence of two symbols, and children age 4–5 had the capacity to memorize three to four symbols (the symbols range was from 0 to 5, (U = 621.0, p < 0.001)).

## Discussion

The results of the study showed that there was significant improvement of various components of EF during the age period from 3 to 5 years. This involved the ability to follow instructions and switch from one element of the program to another (but not yet from program to program), to increase the span and efficiency of visual-spatial working memory, and to concentrate and maintain attention when performing a monotonous task.

Our data on progressive changes in the studied components of EF by the age of 5 years are consistent with the results of Zelazo’s study (Zelazo, 2006), which demonstrated pronounced difficulties in performing the DCCS (The Dimensional Change Card Sort) test for task switching (cognitive flexibility) in 3-year-old children, and the emergence of the ability to perform this test in most children of 5 years. A more recent study by Zelazo et al. (Zelazo et al., 2013) showed that the development of cognitive flexibility in children from the ages of 3 to 6 correlated with their ability stay focused when performing a flanker test. The authors attributed this result to the children’s development of inhibitory control. In general, they noted an increase in the indicators of both fluid abilities and inhibitory control in children of 3-6 years as they aged, as well as a higher correlation with other indicators of cognitive development in this age group than occurs in the group of 8–15-year-old children.

Our study provided new data on the significant progressive changes in some EF functions in children 3 to 5 years old. The most prominent changes with age were found in the understanding of instructions, and the ability to switch from one element of the task program to another, as well as in the ability to maintain concentration during prolonged monotonous activity. It should be noted that all these abilities are very important for knowledge acquisition and learning. Progressive age-related shifts were also found for inhibitory control, a component of EF associated with the selective cognitive regulation of activity.

In 3-4-year-old children, we found the virtual absence of self-execution of task instructions during their performance of the neuropsychological tasks; that was an age-specific trait. However, in several tests (the Cancellation Test, Spot the Difference Task, and Digit Symbol Coding Task), cooperation with an adult helped the children cope with the task. This observation makes it prudent to include a stage of child-adult joint activity in neuropsychological tools creation for young preschoolers. Another distinguishing feature of the younger group was the pronounced individual variability in the performance of some tests: for example, some children did not master the instructions for the Digit Symbol Coding Task even after a long period of collaboration with an adult, whereas other children quickly grasped the algorithm and kept at it until the end of the task, performing it at a fast pace and without errors.

Immature voluntary control of cognitive activity in the younger group allows us to conclude that teaching 3-4-year-old children to write and read is meaningless, since it requires an appropriate level of working memory, selective and sustained attention, and the ability to program one’s activity. Our results are in accordance with the viewpoint of researchers who subscribe to activity theory.

Despite their progress in EF by 4–5 years, both the younger and older groups demonstrated difficulties when performing more complex cognitive operations — for example, switching from one mode (program) of task performance to another — while there were no significant differences between age groups. Along with the results above, these data point to different rates of development (heterochrony) of various components of voluntary regulation of cognitive activity at preschool age.

It should be noted that all the children included in our study demonstrated lower levels of voluntary control than 6-7-year-old children (Zakharova et al., 2022). All the children were struggling with understanding instructions or algorithms (all the children required adult guidance for most of tasks) and had difficulties in maintaining a program which involved monotonous activity (the children were quickly exhausted due to the large number of stimuli), switching algorithms, and self-control.

Changes in age-related working memory (WM) are of particular interest, as it is exactly the retention of relevant information that is directly responsible for concept formation during learning. According to our results, 4-5-year-olds (compared to 3-4-year-olds) significantly increased their working memory span and capacity. These data complement the data on age-related transformations of WM in children age 4 to 15 years, presented in the work of Gathercole et al. (2004).

Thus, the results of our study prove the importance of applying the methodical principles of activity theory to the development of cognitive processes in preschoolers. For instance, it is of great importance to attract the attention of children and direct them to the required task (or switching between tasks), maintaining conditions that ensure the children’s interest in the subject and their attention when performing joint activities. On the other hand, it is also important to teach children methods to develop task orientation and self-control. According to L. Vygotsky’s concept of the *zone of proximal development* (Vygotsky, 1991*)*, active child-adult social interaction could improve voluntary forms of cognitive activity and better prepare a child for systematic education.

Our results suggest that the EF components important for the formation of concepts (general activity skills) are still immature in 3–5-year-old children. According to N.F. Talyzina, these components should be purposefully developed by means of active child-adult social interactions, including the stages of orientation, execution, control, and correction (Talyzina, 1988). In practical terms, this process could be organized as follows. Th\e education of preschoolers should be based on their interests, leading to an increase in motivation. Adults should also help the children go through the orientation and control stages. The orientation stage is important for drawing the children’s attention to the aspects underlying efficient task performance. The control stage is important for organizing the children’s attention to checking their results.

According to the theory of gradual mastering of mental processes, which facilitates the activity approach to the analysis of learning processes (Galperin, 1966), teachers should move from the most expanded, element-by-element action, shared by the child and the adult, to internalized action. It is possible to offer a large number of methodological techniques (for formulating and/or organizing tasks) based on this principle. For example, you can teach a child to act according to the rules as follows. At the first stage, you must put each task (or game) in a separate box. Each box has its own number (usually from 1 to 3–4). This procedure helps the child to move sequentially from one task to another by performing physical actions (the child takes the task out of the box and puts it back after completion). At the last stage, the child uses a card with a plan, where tasks are indicated by pictograms (icons). Thus, the activity approach as a whole and the principles of the formation of cognitive activity, described in the works of N.F. Talyzina, open up great prospects for developing voluntary control in young preschoolers.

## Conclusions

1. Neuropsychological examination of preschool children revealed a heterochrony in the development of various components of voluntary control in children from 3 to 5 years of age.
2. Some executive functions — namely, the ability to assimilate instructions and switch from one element of the program to another, visual-spatial working memory, and the ability to focus and maintain attention when performing a monotonous task — exhibited significant progressive changes from 3–4 to 4–5 years. At the same time, these functions were still immature in both age groups compared to older children, and children of both age groups showed difficulties in switching from one algorithm of action to another, and insufficient voluntary control over their own activity.
3. A specific aspect of voluntary control in 3–5 year-olds is an adult-aided task acquisition, which dramatically increased the efficacy of task completion.
4. The results indicate the importance of the application of the activity theory approach to the development of cognitive processes in preschool age.
